# Identification of Specific Joint-Inflammatogenic Cell-Free DNA Molecules From Synovial Fluids of Patients With Rheumatoid Arthritis

**DOI:** 10.3389/fimmu.2020.00662

**Published:** 2020-04-28

**Authors:** Cong Dong, Yu Liu, Chengxin Sun, Huiyi Liang, Lie Dai, Jun Shen, Song Wei, Shixin Guo, Kam W. Leong, Yongming Chen, Lai Wei, Lixin Liu

**Affiliations:** ^1^Key Laboratory for Polymeric Composite and Functional Materials of Ministry of Education, Center for Functional Biomaterials, School of Materials Science and Engineering, Sun Yat-sen University, Guangzhou, China; ^2^State Key Laboratory of Ophthalmology, Zhongshan Ophthalmic Center, Sun Yat-sen University, Guangzhou, China; ^3^Sun Yat-sen Memorial Hospital, Sun Yat-sen University, Guangzhou, China; ^4^Department of Rheumatology, General Hospital of Guangzhou Military Command of PLA, Guangzhou, China; ^5^Department of Biomedical Engineering, Columbia University, New York, NY, United States

**Keywords:** autoimmunity, cell-free DNA, CpG-motif, inflammation, rheumatoid arthritis

## Abstract

Elevated cell-free DNA (cfDNA) levels in the plasma and synovial fluid of rheumatoid arthritis (RA) patients are proposed to be pathologically relevant. However, direct evidence to support this perception is lacking, and molecular feature of the cfDNA molecules with assumed pathological function is not well characterized. Here, we confirm remarkably increased levels of total synovial fluid and plasma cfDNAs in a large cohort of patients with rheumatoid arthritis compared to the counterparts in osteoarthritis, and demonstrate the potent inflammatogenic effects of RA synovial fluid cfDNA on both human monocyte cell line and primary cells related to RA. Massively parallel sequencing identifies distinct molecular pattern of cfDNA in RA, as characterized by enriching CpG-motif containing sequences. Importantly, these identified CpG-motif-rich sequences are hypomethylated in RA patients and induce severe inflammatory responses both *in vitro* and *in vivo*. Our data demonstrate the pathological role of global and specific cfDNA molecules in RA, thereby identifying novel therapeutic target candidate and potential biomarker for RA.

## Introduction

In pathological conditions, including cancers, inflammatory disorders and severe trauma (1–3), abnormal amounts of cfDNA are released into body fluids following exorbitant apoptosis, necrosis, NETosis, and pyroptosis (4). Some of previous studies of autoimmune diseases, especially in rheumatoid arthritis (RA) and systemic lupus erythematosus (SLE), have shown that increased level of cfDNA in plasma correlates with disease progression, and thus, cfDNA has been used as a marker to monitor therapeutic responses to certain autoimmune diseases (5, 6).

In addition to the application as a biomarker, prior studies have attempted to determine if cfDNA plays a role in inflammation associated with autoimmune diseases (7). Initially, it was shown that vertebrate cfDNA failed to induce an inflammatory response in autologous plasmacytoid dendritic cells, which are sensitive to bacterial nucleic acids. As a result, it was thought that because cfDNAs are products of self-DNA, the host immune system has tolerance to this self-component. However, the development of improved technology led to the finding that cfDNA, especially derived from mitochondrial DNA, has a high degree of oxidative damage and is similar to bacterial DNA, and can bind with LL-37, HMGB, auto-Ig, and other proteins to form immunocomplexes. These immunocomplexes deliver various cfDNAs into cells, which are then recognized by pattern recognition receptors (PRRs), such as TLR-9 or STING, leading to downstream signaling cascade amplification and cytokine overexpression. Consequently, cfDNA contributes to the etiology of psoriatic arthritis, atherosclerosis, and SLE (8–10).

Like SLE, accumulating evidence suggests that cfDNA might play an important role in the pathogenesis of rheumatoid arthritis (RA). For example, intra-articular injection of CpG ODNs, the unmethylated CpG oligodeoxynucleotides prevalent in bacteria, triggers reactive arthritis in mice (11–13). Further, DNase knock-out mice spontaneously develop polyarthritis with symptoms resembling that of human RA (6, 14). Activation of TLR-9 by chromatin combined with IgG2a can stimulate B cells to produce autoantibodies, known as rheumatoid factors (15). Moreover, it is known that the concentration of cfDNA in plasma and synovial fluid (SF) of RA patients is significantly elevated (16–18). However, the direct evidence that endorses the pathological role of cfDNA in RA is not established, and the molecular nature of RA cfDNA potentially implicated in onset and development of RA is unknown.

In this study, we analyzed cfDNA isolated from the synovial fluid (SFcfDNA) of 113 RA and osteoarthritis (OA) patients via massively parallel sequencing and other *in vitro* and *in vivo* approaches. Our findings confirm the inflammatogenic property of cfDNA from RA patients, and the molecular characteristics of SFcfDNA identified in this study provide novel insights into the role of cfDNA in RA.

## Materials and Methods

### Study Design

Plasma and synovial fluid were collected from 163 individuals, including 50 healthy donors (HDs) for plasma, 33 OA subjects for synovial fluid, 80 rheumatoid arthritis patients for plasma and synovial fluid, and the detailed information of the study population is illustrated in Supplementary Table 1. Primary synovial fluid mononuclear immune cells (SFMICs) and fibroblast-like synoviocytes (FLSs) were collected from RA synovial fluid and synovium tissue, respectively, which were stimulated with total cfDNA purified from the SF of the same patient. Intracellular TNF-α staining and cytokine bead assay (CBA)/ELISA were carried out for detection of cytokine expression. Then, the cfDNA size distribution frequency and the discrepancy sequence feature between RA and OA were analyzed. Specific CpG-motif-rich (CMR) sequences and CpG-motif-free (CMF) sequences were acquired by virtue of bioinformatics technique, which would be described in detail in the method of sequencing and analysis. Finally, pro-inflammation capability of high-frequency sequences was tested *in vitro* and *in vivo*.

### Participants

All the plasma, synovial fluid, and synovium samples of RA patients were collected from Sun Yat-sen Memorial Hospital, General Hospital of Guangzhou Military Command of PLA after informed consent. RA patients who were selected for the study were confirmed with ACR 2010 standards, without bacterial and virus infection. SF of OA control was collected from patients who underwent arthroplasty surgeries in the First Affiliated Hospital, Sun Yat-sen University. Healthy plasma samples were obtained from volunteer donors. The average age of RA (*n* = 80), OA patients (33) or HDs (*n* = 50) was 56 ± 3, 58 ± 4, 48 ± 5, respectively. For the sequencing investigation, the subjects were female with the average age 48.5 (±12.7) years in the RA group and 51.0 (±6.2) years in the OA group. The detailed information of patients used in each experiment are displayed in Supplementary Table 1. Studies were approved by the ethics committee of the General Hospital of Guangzhou Military Command PLA, Sun Yat-sen Memorial Hospital and the First Affiliated Hospital of Sun Yat-sen University, respectively. The recruited patients gave written consent according to a protocol approved by the above Committees.

### cfDNA Purification and Quantification

After synovial fluid was extracted from joints of patients, an equal volume of PBS was added to dilute the samples, followed by adding hyaluronidase (1 mg/ml, pH = 7.4) and incubated at 37°C for 0.5 h. Then Ficoll-Paque was used for mononuclear cell sorting, and supernatant was collected for cfDNA purification. cfDNA was purified with circulating cfDNA purification kit following the instructions of the manufacturer. The purified cfDNA concentration was quantified via Picogreen^@^ dye.

### Intracellular TNF-α Staining Assay

After diluting SF with PBS, the samples were incubated with hyaluronidase (1 mg/ml, pH = 7.4) at 37°C for 0.5 h. Then the samples were passed through a 70-μm filter, and the SFMICs were collected with Ficoll-Paque. Isolated SFMICs were plated in 24-well plates at a density of 5 × 10^5^ cells/ml with RIPM-1640 (Gibco) completed medium and transfected with 500 ng of cfDNA or CpG 2006 for 24 h via Lipofectamine 2000 (Lipo2000; Invitrogen) following instructions of the manufacturer. The wells with equal volume of Lipo2000 were used as background controls. Monensin solution (1:1,000; BioLegend) was added to inhibit the cytokine secretion before sample staining. After the cells were blocked with Human TruStain FcX^TM^ kit (1:50; BioLegend), they were stained with AF700 Human CD45 (1:100; BioLegend) (Supplementary Table 2) for 30 min at room temperature. Viability was determined by Zombie Yellow^TM^ Fixable Viability kit (1:1,000; BioLegend). Cells were washed twice with PBS, fixed and permeabilized with Transcription Factor Fix/Perm Buffer (eBioscience). After the cells were washed by PBS with 2% FBS, they were stained with BV421 Human TNF-α (1:100; BioLegend) (Supplementary Table 2) for 30 min. Then the samples were analyzed by Attune CytoFlex analyzer (ThermoFisher). For TNF-α staining of THP-1 cell line, the same procedure was performed, except for CD45 staining. Briefly, a percentage of TNF-α-expressing cells was analyzed through flow cytometry. Gating strategy was based on, first, viable cells gating via Live/Dead dye, followed by CD45^+^ gating, then singlet cells of CD45^+^ cells were gated according to FSC-A/FCS-H properties. Finally TNF-α positive cells were determined.

### Cytokines Expression

#### FLS Isolation

Synovial samples were acquired from discarded arthroplasty tissue. After careful dissection, mincing, and digestion with Collagenase I (1 mg/ml; Sigma-Aldrich) in RIPM medium (Gibco) for 30 min with agitation, cells were passed through a 70-μm cell strainer. Then the cells were subjected to red cell lysis and were cultured with DMEM high-glucose completed medium. Three- to nine-passage primary FLS was used for functional assay. Primary FLS validation: Primary FLS used for the study was measured by flow cytometry. The percentage of double positive ratio of PE Anti-Human CD55 (1:100; BioLegend) and PerCP-Cy5.5 Anti-Human CD90 (Thy1; 1:100; BioLegend) (Supplementary Table 2) was more than 98%. For stimulation assay, 500 ng of SFcfDNA was transfected to FLS at a density of 5 × 10^3^ cells per 300 μl in 48-well plates via Lipo2000 following manufacturer's instructions, and an equal volume of Lipo2000 (1 μl) was added into the control wells. After 4 h, the medium was changed, and after 24 h, the supernatant was collected for cytokine evaluation with human ELISA kit Ready-SET-Go! (eBioscience). The concentration of cfDNA for FLS stimulation assay without transfection was 15 μg/ml. For SFMIC stimulation assay, 1 × 10^5^ cells were seeded into 96-well plates, and 500 ng of SFcfDNA was transfected to cells with Lipo2000. After 24 h, the supernatant was collected for cytokine evaluation with ELISA kit. High-frequency sequence PCR purification and amplification were obtained with Advantage® GC2 PCR kit (Takara Biomed), PrimeSTAR® Max (Takara Biomed) on Mastercycler nexus X2 PCR platform (Eppendorf), QIAquick Gel Extraction kit (Qiagen), and QIAquick PCR Purification kit (Qiagen). For CMRs and CMFs stimulation to THP-1 cell lines, the same process was performed as that of SFMIC.

### Illumina Sequencing

Cell-free DNA was extracted from SF samples using the MasterPure Complete DNA and RNA Purification kit (Epicenter) according to the instructions of the manufacturer. Next, the sequencing library was prepared using the ND604-VAHTS Universal DNA Library Prep kit (Vazyme) based on the protocol. Then the DNA library was sequenced on Illumina HighSeq 2500 sequencer, and the initial image processing was done by CASAVA (v1.8.2, Illumina).

### Sequencing Data Analysis

Sequencing reads were cleaned using Trimmomatic (version 0.36) (19) in order to (i) remove the primers and sequencing adapters; (ii) trim nucleotides with the average quality per base drops below 15 in a four-base-wide sliding window from the 3′ end; and (iii) drop read below the 30 bases long. Cleaned reads were subsequently aligned to the human genome (hg19) using BWA (version 0.7.15) (20). The reads that were uniquely mapped to the human genome were retained for analysis. To compare the genomic distribution of SFcfDNA between samples, two types of fragments were investigated and a normalized sequence density for each fragment, which is similar to the RPKM (Reads Per Kilobase Million) in RNA-seq analysis, was calculated as follows:

$$\begin{array}{l} \text{Sequence density}=\frac{\text{number of reads mapped to a CpG sequence}}{\begin{array}{c} \text{Sample}-\text{specific normalized factor }\times \\ \text{CpG length }\left(50\text{ kilobase}\right) \end{array}} \end{array}$$

The first type of fragments was chromosome bins with 400-bp width, which provided genomic distribution of SFcfDNA molecules, and the other was the CpG islands across the human genome, which is epigenetically relevant. The sample-specific normalized factor is used for sequencing depth normalization. In detail, a sliding window of 50 kilobase was applied across each chromosome, and the number of sequences falling within each window was counted, and the median value was chosen to be the representative of the chromosome. Because the sex chromosome for each sample might be different, the median sequence representatives among autosomes were used as the normalized factor. Human genome sequence (hg19) and CpG positions from hg19 were downloaded from the UCSC website (http://genome.ucsc.edu/) on April 12, 2018. In total, 28,691 CpG sequences were computationally annotated. Because all the participants in the sequencing cohort were female, CpG islands in human chromosome 1–22 and X, which included 27,537 CpG positions, were involved in the analysis. All the read numbers were counted by featureCounts (version 1.5.2), and the normalized sequence densities were used for comparison among samples in the CpG-enrichment analysis (21).

### Total cfDNA and CMR Methylation

For detection of the global SFcfDNA methylation levels of RA and OA, we applied the 5-mC DNA ELISA kit (Zymo Research) according to the manufacturer's instructions. In brief, 100 ng of DNA was coated on the wells of the plate. Then primary (specific for 5-methylcytosine) and secondary antibodies were added to the wells. The OD value was read out by a Bioteck microplate reader at a wavelength of 450 nm. The result was calculated via a standard curve generated by different percentages of methylation levels of standard 5-mC DNA. Specific CMR methylation in RA and OA SFcfDNA was determined with a OneStep qMethyl-Lite kit (Zymo Research). Briefly, 20 ng of global DNA was incubated in the presence (test reaction) or absence (reference reaction) of methyl-sensitive restriction enzymes (5 U each; BStUI, HpyCH4IV and HpaII, NEB) at 37°C for 2 h, followed by real-time reverse transcription PCR (RT-PCR) as described in the manufacturer's instructions. Percentage methylation was calculated using the formula 100 × 2^−Δ*Ct*^, where ΔCt is the average Ct value from the test reaction minus the average Ct value from the reference reaction. Percentage methylation is relative to each experiment.

### SiRNA Knock Down Assay

Three different sequences of human TLR-9 and siRNA were designed and ordered from RiboBio Biotechnology, Guangzhou, China, and are listed in Supplementary Table 3. SiRNAs (200 nM) were transfected via Neon Transfection System (Life technology, Thermo Fisher) following the manufacturer's instruction. The down-regulation of target genes was evaluated by qPCR, with GAPDH as the internal control. After 24 h of stimulation, cell supernatants were collected for TNF-α and IFN-β detection.

### Animal Assay

Female BALB/c mice (6–8 weeks) were purchased from Guangdong Medical Laboratory Animal Center. All mice were bred, housed, and used under specific pathogen-free conditions in the animal facility of the School of Life Science, Sun Yat-sen University. The animal studies were performed with the approval of the ethics committee of the School of Life Science, Sun Yat-sen University. At 1 day, 6 μg of CMR-3, CMR-7, CpG 1668 (positive control), CMF-4 in 20 μl of sterile PBS and the same volume of PBS were intra-articularly injected into mouse knees. The same dose of DNA was boosted at Day 4, respectively.

### Joint Swelling Measurements and Clinical Scores

The diameter of mice articular joints was measured by a vernier caliper (Guanglu) from the first day of the onset of arthritis. The clinical scores were acquired according to the reported standard description (22): Score 0: no evidence of erythema and swelling occurred. Score 1: erythema and mild swelling appeared. Score 2: erythema and mild swelling extended from the ankle to the tarsals. Score 3: erythema and moderate swelling extended from the ankle to metatarsal joints. Score 4: erythema and severe swelling encompassed the ankle, paws, and digits or ankylosis of the limb.

### Morphology Study

#### Photograph

After articular injection of DNA sequences at 3 days, the hindpaws of mice were photographed by a camera. Micro-CT imaging: At day 6, the mice were sacrificed, and the joints were fixed in 10% buffered formalin for a week, then scanned at 80 kV and 500 μA with the resolution of 19 μm in *ex vivo* micro-computed tomography (Siemens Inveon Micro-CT/PET) for 50 min. Three-dimensional images of joints were obtained by Inveon Research Workplace. Bone mineral density and the trabecular parameters including bone volume/total volume, bone surface area/bone volume, trabecular thickness, trabecular spacing, and trabecular pattern factor were measured by Inveon Research Workplace. MRI imaging: The mice were anesthetized by i.p. injection of 3.5% chloral hydrate, and then the Coronal fat-suppressed T2W TSE MRI (MRI; Achieva 3.0T; Philips Healthcare) of the inflammation articular joints were obtained with an eight-channel transversal animal coil. The parameter briefly: time of repeat/time of echo, 800/80 ms; field of view, 55 × 55 mm^2^; slice thickness, 1 mm; slice number, 8; number of signal average, 1; matrix, 176 × 190. Spectral pre-saturation inversion recovery (SPIR) fat suppression technique was used. The total imaging time of this sequence was about 4 min 19 s. Readers scored synovitis, bone marrow edema, and bone erosions according to the Rheumatoid Arthritis MRI Scoring (RAMRIS) system (23). RAMRIS scores of mouse joints were obtained following a standard evaluation process. The scale of synovitis is 0–3. Score 0–4 is normal, mild, moderate, severe, respectively. The scale of bone marrow edema is 0–3. 0 = no osteitis; 1 = 1–33% of bone with osteitis; 2 = 34–66%; 3 = 67–100%. The scale of bone erosions is 0–10. 0 = no erosion; 1 = 1–10% of bone eroded; 2 = 11–20%; 3 = 21–30%, etc. The cumulative score of synovitis, bone marrow edema, and bone erosions on a scale of 0–16 served as a measure of the severity of inflammation and joint damage.

### Histological Analysis and Immunohistochemical Staining

At day 6, the animals were sacrificed, then the ankle, knee, digital, and wrist joints were fixed with 10% buffered formalin. After, joints were incubated in decalcifying solution (4% hydrochloric acid in 4% formaldehyde) at room temperature for 7 days. The samples were paraffinized and cut into microtome (Leica) slices of 2 μm for histological examination. After staining the slices with hematoxylin and eosin, respectively, the inflammatory cell accumulation in synovial tissues, bone, and cartilage was evaluated by Vectra Automated Quantitative Pathology Imaging System (PerkinElmer).

To evaluate the histology scores of the knee and ankle joints of each mice, the histopathologic feature was graded using a scoring system as previously described (24): synovial cell lining hyperplasia (0–2); mononuclear cell infiltration (0–3); pannus formation (0–3); polymorphonuclear leukocyte infiltration in periarticular soft tissue (0–3); cellular infiltration and bone erosion at distal tibia (0–3); and cellular infiltration of cartilage (0–2). In addition, the sum of all the histopathologic feature scores was the histology score of each animal joint.

### Real-Time PCR

At day 6, the mice were sacrificed, then the tissue just above the ankle joint with shinbones were removed and homogenized in TRIzol^TM^ reagent with a homogenizer (T18 digital ULTRATURRAX, IKA) as described previously (6). Briefly, total RNA was extracted with Rneasy Universal Tissue kit (Qiagen); then, they were reverse-transcribed into cDNA using PrimeScript^TM^ RT Reagent kit with gDNA Eraser and oligo (dT; Takara Biomed) and SYBR® Premix Ex Taq^TM^ II kit (Takara Biomed). qPCR was carried to compare the cytokine expression, and glyceraldehyde-3-phosphate dehydrogenase (GAPDH) was the internal control. The relative mRNA levels of cytokines and MMP-3 in the normal group were set as 1. The sequences of the primers are shown in Supplementary Table 3.

### Statistical Analysis

Statistical differences were calculated with PRISM 5.0 software (GraphPad) using non-parametric Mann–Whitney test, two tailed-paired T test, one-way ANOVA, and Kruskal–Wallis Dunn's multiple comparison. Data were considered statistically significant when values were *P* ≤ 0.05.

## Results

### Increased cfDNA Levels in Large Cohort of RA Patients

**Figure 1:**
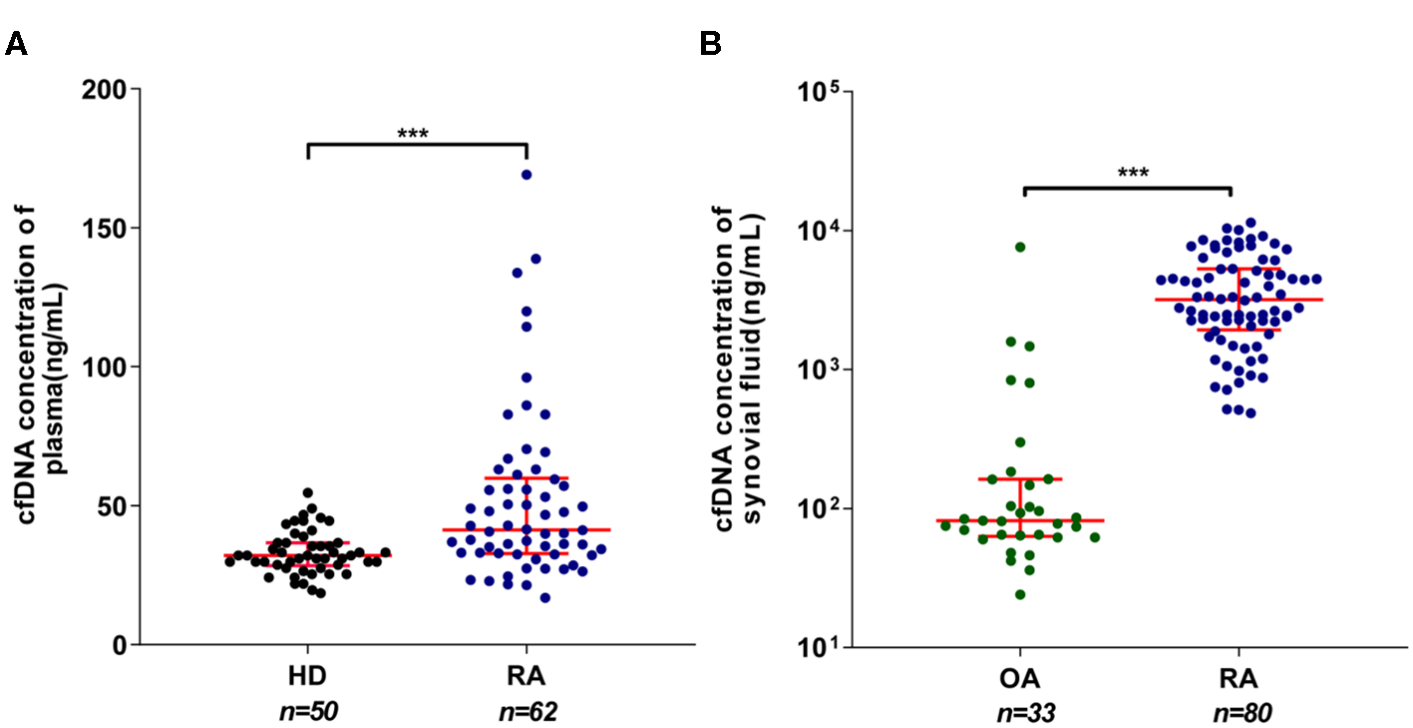
Elevated cell-free DNA (cfDNA) isolated from the synovial fluid (SFcfDNA) concentrations in RA patients. **(A)** cfDNA concentrations in plasma of healthy donors (HDs) and rheumatoid arthritis (RA) patients. **(B)** cfDNA levels of synovial fluid in RA and osteoarthritis (OA) patients. Data was calculated by non-parametric Mann–Whitney test; *P* < 0.05 is recognized as statistically significant. ^***^P < 0.001. Data are presented as median with interquartile range.

A number of prior studies reported elevated cfDNA levels in circulation and SF of RA patients (16, 25). However, contradictory data were also emerged recently (26). To clarify this issue, we evaluated the concentrations of plasma and SF cfDNA in large control and RA patient population (80 RA cases) via Picogreen-based assay that only detects intact dsDNA. Our data show that there was a significant difference in plasma cfDNA levels between patients and healthy individuals (median: 41.3 vs. 32.1 ng/ml) (Figure 1A), and the median SFcfDNA level in RA patients was 38.8-fold higher than that in OA individuals (3,182 vs. 82 ng/ml) (Figure 1B). Furthermore, the median cfDNA concentration in the SF was ~77-fold higher than that in the plasma in RA (3,182 and 41.3 ng/ml, respectively). These data confirm increased plasma and substantially high SF cfDNA levels in the relatively larger RA patient cohort (80 cases), and the fact that super high cfDNA concentrations are anatomically restricted within joints strengthens the proposal that cfDNA may be implicated in onset and development of RA.

### Cellular Stimulatory Function of SF cfDNA From RA Patients

**Figure 2:**
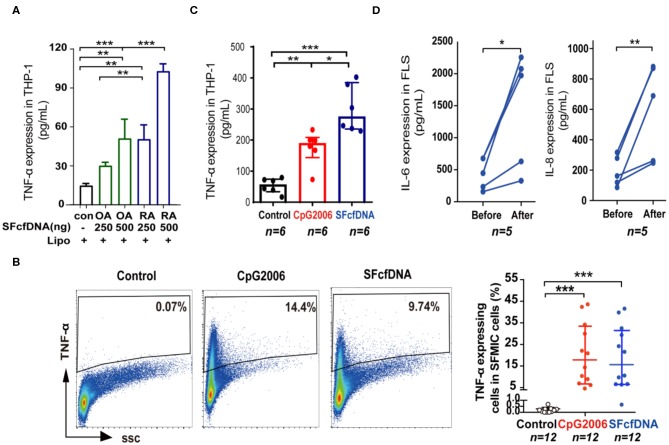
Cellular stimulatory function of SFcfDNA from RA patients. **(A)** Expression of TNF-α by THP-1 cells stimulated with SFcfDNA from OA or RA patients at two concentrations. The data shown are representative of three independent experiments with the same tendency. **(B)** The percentage of primary SFMICs from RA patients expressing intracellular TNF-α upon stimulation with SFcfDNA or CpG 2006. The data on the left is a representative sample and on the right is the mean of 12 samples. **(C)** TNF-α secretion by SFMICs (*n* = 6) stimulated with CpG 2006 or SFcfDNA (0.5 μg/2 × 10^5^ cells) using the same procedure. **(D)** IL-6 and IL-8 expression on primary FLSs from RA patients (*n* = 5) stimulated with SFcfDNA from RA patients. Statistical significance was calculated by one-way ANOVA **(A)**, Kruskal–Wallis Dunn's multiple comparison test **(B,C)**, two-tailed paired T testing **(D)**. *0.01 < *P* < 0.05, **0.001 < *P* < 0.01, ***P < 0.001. Data are presented as the mean with SD **(A)**, and **(B,C)** data are presented as the median with interquartile range.

To further define the pathological relevance of SF cfDNA, we determine if SFcfDNA can induce inflammatory response. SFcfDNA isolated from both RA and OA patients was used to stimulate THP-1 cells, a human monocyte cell line, which express TLR-9 and STING DNA sensors via transfection. Owing to the high variation in SFcfDNA concentrations seen in OA patients (24 ng/ml−1 μg/ml), we pooled synovial fluid from 10 separate OA cases to obtain enough cfDNA to stimulate inflammatory cells, and from 10 separate RA cases as a compared group. Results indicated that SFcfDNA from both RA and OA patients can stimulate THP-1 cells to produce inflammatory cytokine (TNF-α). SFcfDNA from RA patients was even more potent at low doses (250 ng/2 × 10^5^ cells) (Figure 2A). Moreover, SFcfDNA from RA patients could stimulate primary SFMICs (Supplementary Figure 1A) and FLSs (Supplementary Figure 1B) to produce pro-inflammatory cytokines. Briefly, flow cytometer analysis showed that an average of 13.8% of SFMICs expressed TNF-α after SFcfDNA stimulation (Figure 2B), which was further confirmed by measuring TNF-α expression in SFMIC culture supernatant by ELISA (Figure 2C). Moreover, expression of IL-6 and IL-8 was elevated after SF cfDNA challenging FLSs (Figure 2D). However, no increased expression of IL-1β, TNF-α, or IFN-α was observed with FLSs, even present at a dose as high as 1 μg/ml (Supplementary Figure 1C). Thus, our results directly demonstrate the flammatogenic property of RA global SF cfDNA *in vitro*.

### Genomic Landscape and Molecular Feature of SFcfDNA From RA

**Figure 3:**
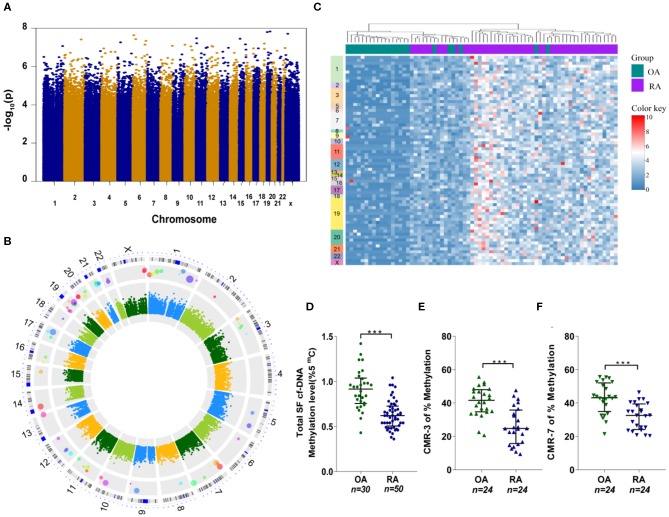
SFcfDNA distribution and CpG-motif-rich (CMR) sequences in synovial fluid. **(A)** Distribution of total cfDNA density across chromosomes. Briefly, Manhattan plot with –logarithm *P*-values for the distribution comparison of total cfDNA density across chromosomes. Sequence density of 400-bp chromosome bins across the human genome was used for the measurement of overall genomic distribution of SFcfDNA molecules. Totally 7,590,771 chromosome bins were included and compared. **(B)** Circular Manhattan plot for the distribution comparison of SFcfDNA at CpG-motif regions. Features aligned onto the main chromosomes as concentric rings (from inner to outer) correspond to (1) Between-group difference (RA–OA) of sequence density of CpG-island regions. The values were coded by color, and the key legend was shown in the center. (2) Manhattan plot with –logarithm *P*-values for the comparison of sequence density of CpG regions. Totally 28,691 CpG sequences were compared, and the Bonferroni-corrected cut-off with minus logarithm scale is about 5.8. (3) CpG-rich locations identified by sequence density comparison at CpG regions. The larger the circle is, the higher significance it has. (4) The chromosome ideograms. (5) The number of chromosomes. **(C)** The average sequence density in every CpG location grouped by RA (purple) and OA (green), which clearly shows the presence of CMR sequences mainly in RA-cfDNA originated from RA patients. **(D)** Assessment of global cfDNA methylation levels in RA (*n* = 50) and OA (*n* = 30) patients through analysis of the percentage of 5-mC by ELISA. **(E,F)** Percentage of methylated **(E)** CMR-3 and **(F)** CMR-7 in SFcfDNA from RA and OA patients. **(D–F)** Statistical significance was calculated by non-parametric Mann–Whitney test, ***P < 0.001. Data are presented as median with interquartile range.

To identify and characterize individual inflammatogenic SFcfDNA molecules, we investigated the genomic distribution and sequence features of SFcfDNA from RA (*n* = 50) and OA (*n* = 23) patients using whole-genome shotgun sequencing (27). The patients were all female with an average age of 48.5 (±12.7) years old in the RA group and 51.0 (±6.2) years old in the OA group (Supplementary Table 1). Sequencing generated a total of 3.7 billion paired-end 125-bp reads, of which 94.9% (median) passed quality control. Of these post-QC reads, 95.4% (~42.3 million per sample) on average aligned uniquely to human genome (Supplementary Figure 2). The number of sequencing reads for each sample is listed in Supplementary Table 4. Uneven distribution of reads across each chromosome was observed, and this intra-chromosomal variation was common among all samples, likely due to sequencing artifacts. However, the number of reads mapped to each 50-kb sliding window per chromosome in each of the samples remained consistent among all chromosomes. The sample-specific normalized factors were calculated using the number of reads per 50-kb sliding window to account for the differences in total sequencing yields among samples. Sequence densities of different regions in all the samples were analyzed. Overall comparison of sequence densities across the whole genome did not show any distinct regions with deeper coverages in the RA group (Figure 3A). However, the analysis, focused on epigenetically relevant CpG-island regions, revealed that the majority of these sequences had higher sequence densities on average in RA patient samples compared to the counterparts in OA patients. More specifically, 71 CpG-motif-rich (CMR) sequences were identified using the Wilcoxon rank-sum test for between-group comparison with Bonferroni-corrected alpha level (Figure 3B), and can be classified into two subgroups, CpG-motif-rich sequences with equal to or >20 CpG dinucleotides (CMR^high^) and CpG-motif-rich sequences with <20 CpG dinucleotides (CMR^low^). These CpG-islands were distributed across every chromosome, although there were more in chromosomes 1 and 19. More importantly, one-way clustering plot showed that RA and OA patients could be separated by the frequencies of these 71 CMR sequences, which could be potentially used as biomarkers to distinguish between OA and RA (Figure 3C).

Considering that hypomethylated DNA sequences (like mitochondrial DNA and bacterial DNA) are believed to induce a greater inflammatory response (28, 29), we next compared the total methylation levels of SFcfDNA between RA (*n* = 50) and OA (*n* = 30) patients. The global methylation level of SFcfDNA in RA patients was found to be significantly lower than that in OA patients (Figure 3D). Next, we randomly chose two specific CMR^high^ sequences, CMR-3 and CMR-7, to compare their methylation statuses in SFcfDNA between RA and OA patients (both *n* = 24). The results showed that both of the sequences were significantly more hypomethylated in RA patients compared to those in OA patients (Figures 3E,F).

Taken together, these results provide the first evidence that SFcfDNAs in RA are enriched with specific CMR sequences, which are hypomethylated.

### Specific SFcfDNA Molecules Induce Potent Proinflammatory Effects *in vitro*

**Figure 4:**
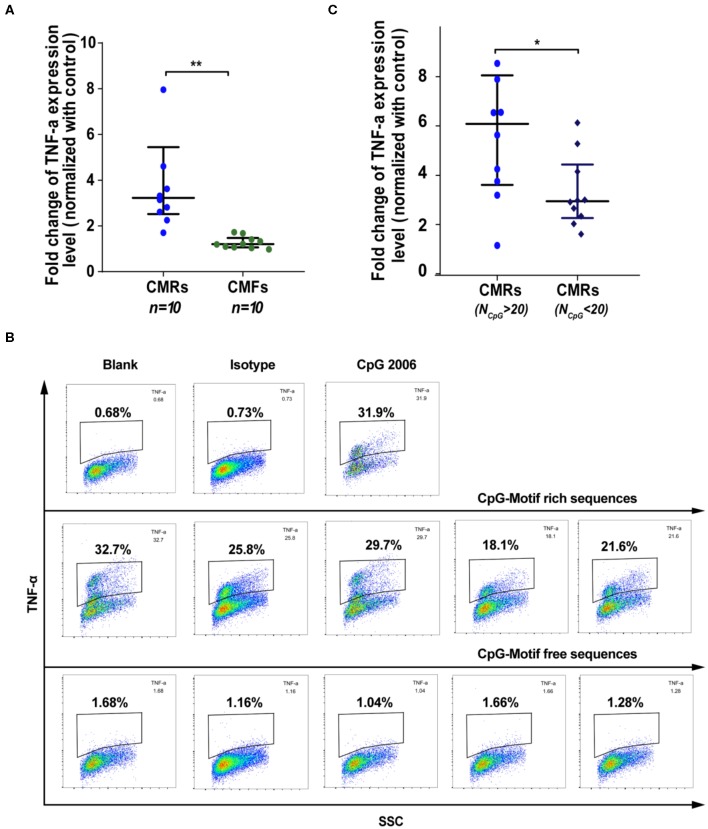
SFcfDNA with CpG motif-rich sequences from RA patients induces TNF-α expression in THP-1 cells. **(A)** TNF-α regulation after stimulation with CMRs and CpG-motif free (CMFs) (250 ng/2 × 10^5^ cells, respectively). Fold change of THP-1 expression was normalized against the control group. **(B)** Percentage of THP-1 cells expressing TNFα expression following stimulation of CMRs from SFcfDNA. **(C)** TNF-α expression induced by CMRs containing different numbers of CpG groups. **(A,C)** Statistical significance was calculated by non-parametric Mann–Whitney test, *0.01 < *P* < 0.05, **0.001 < *P* < 0.01. Data are presented as median with interquartile range.

Hypomethylated CpG-motif-rich (CMR) sequences were previously shown to have a strong pro-inflammatory capability (30, 31), and epigenetic changes were reported to occur in RA-related cells (32, 33). Thus, we hypothesized that the 71 CpG-enriched sequences identified in SFcfDNA of RA patients might be responsible for the inflammatory responses induced *in vitro* by total RA SFcfDNA as described above. To test this, we investigated whether RA CpG-motif-rich RA-CMR^high^ could trigger strong inflammatory response in THP-1 cells with RA CpG-motif free (RA-CMF) sequences of the same length served as control. The individually purified CMR and CMF molecules were obtained by amplifying RA SFcfDNA via PCR with specific primers (Supplementary Figure 3 and Supplementary Tables 5, 6). The cell-based analysis showed that the RA-CMR^high^ could robustly stimulate THP-1 cells to express inflammatory mediator TNF-α, with its levels reaching 1.3- to 4.2-fold higher than those induced by RA-CMFs (Figure 4A). Also, intracellular staining revealed a similar finding. Specifically, TNF-α was produced in 25.3% on average of THP-1 cells in the presence of RA-CMR^high^; in contrast, only did 1.36% of RA-CMFs-stimulated THP-1 cells, displaying 18.6-fold difference in the ability of inducing intracellular TNF-α production (Figure 4B). Moreover, among the 71 CpG-motif-rich sequences identified in this study, 10 RA-CMR^high^ sequences induced higher TNF-α expression levels than did nine RA-CMR^low^ sequences (5.9- vs. 3.9-fold) (Figure 4C and Supplementary Table 6). This is consistent with a previous study showing that CpG density correlates with the ability to induce inflammation (31). Next, we explored the signal cascade activation by the RA-CMR sequences in THP-1 cells. Two RA-CMR sequences (CMR-3 and CMR-7), CpG 2006, and one RA-CMF sequence (CMF-4) were used. The results show that CMR-3 and CMR-7 highly up-regulated TNF-α expression as that of CpG 2006 (Supplementary Figure 4A). On the contrary, CMF-4 hardly activated TNF-α expression. In terms of IFN-β expression, all sequences were stimulated at a low extent, and no significant difference was found between CMRs, CpG, and CMF. After knocking down TLR-9 by siRNA, TNF-α induction obviously declined in CMRs and CpG group compared to the wild-type group, while the significant alteration of IFN-β was not observed in all groups (Supplementary Figure 4B). Thus, RA-CMRs mainly induced inflammatory response through the TLR-9 pathway. Since we translocated cfDNA sequences into the cell with Lipofectamine, we need to make sure that the sequences could enter into the endosome and interact with TLR-9. Cy3-labeled CpG 2006 was transfected to the THP-1 cells with Lipo2000, and another known endosomal transfection reagent, DOTAP, was used as control (34, 35). The results show that both transfection reagents can translocate CpG 2006 into the endosome of the THP-1 cell (Supplementary Figure 4C). Overall, analysis of the stimulated human monocytic cell line THP-1 confirms the potent *in vitro* inflammatogenic property of the individual RA-CMR^high^ molecules identified by short-gun genomic sequencing.

### The Specific RA-CMR^high^ Molecules Induce Joint Inflammation *in vivo*

**Figure 5:**
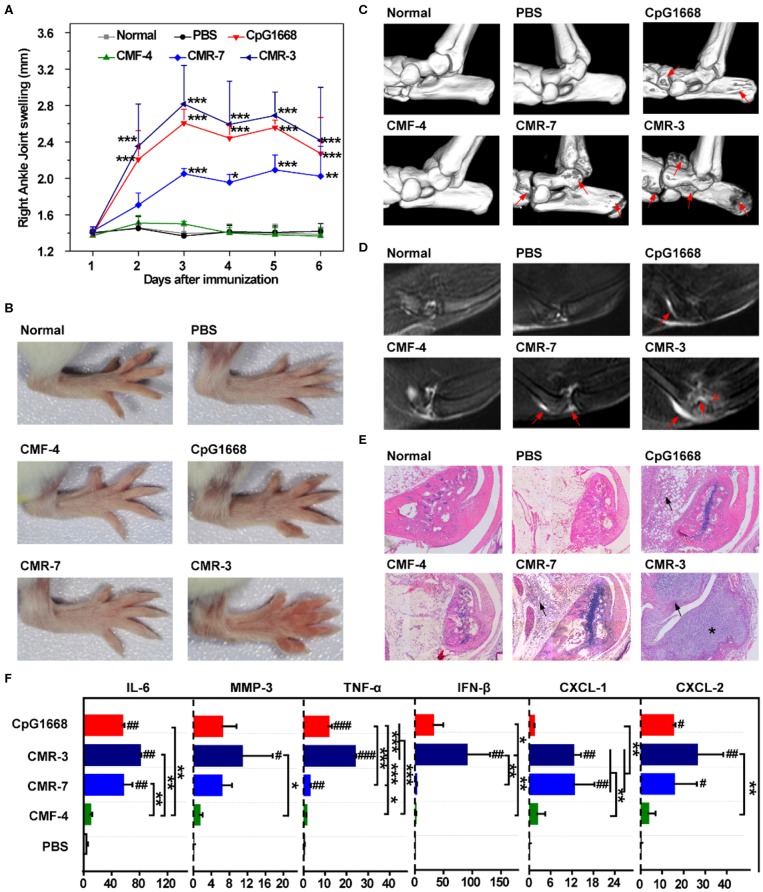
Challenge of BALB/c mice with high-frequency CMR sequences. **(A)** Swelling of the right ankle joint as measured by the change in ankle diameter (*n* = 5). **(B)** Photos of right hindpaws on day 3 after challenge with specific sequences. **(C)** Representative 3D micro-CT images of the ankle joints of mice after administration for 6 days (resolution: 19 μm). Red arrows indicate bone destruction. **(D)** Representative T2-weighted MRI images of ankle joints of different groups at day 6. The effusion in the joint is highlighted by red arrows. Bone erosion in the CMR-3 group is highlighted with a red asterisk. **(E)** Representative HE staining of ankle joints of mice 6 days after administration (×200). Inflammatory cell infiltration in the articular cavity is indicated with black arrows. Bone destruction in mice treated with CMR-3 is highlighted with an asterisk. **(F)** mRNA level of related cytokines and MMP-3 of shinbones at day 6 after immunization. Relative mRNA expression was normalized to β-Actin. **(A,F)** Significance was calculated by one-way ANOVA. ^#^0.01 < *P* < 0.05, ^*##*^0.001 < *P* < 0.01, ^*###*^*P* < 0.001 vs. PBS group. *0.01 < *P* < 0.05, **0.001 < *P* < 0.01, ***P < 0.001 between two groups. Data are presented as mean with SD.

Given that RA-CMR^high^ was shown to induce strong inflammatory responses in THP-1 cells (Figures 4A–C), we next investigated whether these hypomethylated RA-CMR^high^ molecules could induce RA-like joint arthritis in mice. CMR-3, CMR-7, CMF-4, and CpG 1,668 (6 μg/kg, respectively) were injected into the articular cavity of knees in mice on days 1 and 4, as described previously (36). Following the knee joint challenging, assessment of disease status was conducted, including the joint swelling scores (Figures 5A,B), micro-CT examination of bone erosion (Figure 5C and Supplementary Figure 5A), MRI studies of effusion in the knee joints, swelling of the suprapatellar bursa and bone erosion (Figure 5D and Supplementary Figure 5B), histopathological exam (Figure 5E for ankle joints, Supplementary Figure 5C for knee joints, and Supplementary Figure 5D for histology scores), as well as mRNA levels of cytokines in the shinbones (Figure 5F). Similar with what was observed in the positive control (CpG 1668) group, the mice treated with two individual hypomethylated RA-CMR^high^ molecules CMR3 and CMR-7 developed joint arthritis within 3–4 days. This RA-CMR^high^-induced arthritis was characterized by severe joint or paw swelling and joint inflammation with massive leukocyte infiltration and bone erosion, as evidenced by assessing the disease parameters (Figures 5A–F). When evaluated with the same methods, in contrast, mice injected with PBS or CMF-4 had no joint inflammation and bone destruction observed (Figures 5A–F). These findings confirm the inflammatogenic ability of the RA-CMR^high^ but not RA-CMF molecules to induce joint arthritis *in vivo*.

## Discussion

By this work, we further support the observation of abnormal high cfDNA content in the plasma of RA. Moreover, we found that cfDNA content from synovial fluid of RA was much higher than that of OA patients, implying important roles of cfDNA to RA. Previous researches on cfDNA focused on quantitative measurement and biomarker functions, and we have shown that using cationic materials to scavenge cfDNAs may reduce inflammatory symptoms of the RA rat model (36–38). However, the pathogenetic functions of cfDNA in RA remained unclear. Now we showed that SFcfDNA from RA patients elicits robust expression and production of proinflammatory cytokines not only in THP-1 cells but also in primary SFMICs and FLSs from the same RA patients. It is noteworthy that SFcfDNA from OA patients does not have the proinflammatory stimulation ability *in vitro* when applied at the same doses as those from RA patients. Thus, for the first time, the present study provides a direct evidence that, unlike the OA SFcfDNA, the global RA SFcfDNA is inflammatogenic, facilitating the potent induction of inflammatory mediators (TNF-α and IL-6) that are critical for RA pathogenesis. More importantly, through whole genome shotgun sequencing, 71 CpG-motif-rich sequences were found with high frequencies in SFcfDNA from RA patients. One-way clustering plot analysis allows to classify RA and OA patients based on the CpG contents of their SFcfDNA. We found that the unique sequences in SFcfDNA of RA patients are able to mediate the pro-inflammatory cytokine induction. Among the characterized SFcfDNAs, RA-CMR can stimulate greater production of inflammatory cytokines than RA-CMF. Moreover, the immunostimulatory ability of SFcfDNAs correlates with the density of CpG distribution. These *in vitro* results were validated in an animal model where RA-CMR^high^, but not RA-CMF, is able to induce even more severe joint inflammation and bone erosion. As reported previously, hypo-methylated CpG-rich sequences have a strong pro-inflammation capability (30), and bacterial DNA is highly pro-inflammatory because unmethylated CpG motifs are present at the expected frequency of 1 per 16 dinucleotides in the bacterial DNA (31). Also, in the pathological condition of rheumatoid arthritis, epigenetic change has been observed in many studies (32, 33). Therefore, for the first time, with high-resolution analysis, we have demonstrated the characteristic features of cfDNA molecules in RA.

Like RA, SLE is another autoimmune disease, and the elevated cfDNA in plasma of patients was first reported in 1966 (39). Since then, the quantity and quality of cfDNAs in SLE have been studied. For example, high frequency G + C-rich DNA fragments showed more affinity to DNA antibody (40), and drug inhibited T-cell DNA methylation can induce autoimmune syndrome (41). Recently, hypomethylation of cfDNA in SLE was also reported by high-resolution analysis with massive parallel genomic and methylomic sequencing study (42). However, there is still no report to show high inflammatogenic-specific sequences of cfDNA. Therefore, our understanding on qualitative feature exampled by RA case will be also valuable to other cfDNA-induced autoimmune diseases. We may give a hint that there are distinct sequences in total cfDNAs that are relevant to certain autoimmune disease and even cancer. In RA patients, the functions of DNase I in circulation, DNase II in lysosomes, and cytoplasmic TrexI are deficient (6, 43, 44). The abnormal DNAases matter in the digestion of RA patient's genomic DNA, and therefore, the remained sequences of cfDNA in circulation become specific to RA development. The questions like the relationships between these cfDNA fragments and DNAase certainly need to be disclosed.

In conclusion, for the first time, we have demonstrated that the qualitative feature of cfDNA is important for the pathogenic development of RA. We show that high-frequency CMRs in SFcfDNA of RA patients stimulate a strong inflammatory response. Therefore, this work supplies not only a strategy to classify RA and OA patient but also a target of treating RA.

## Data Availability Statement

Our raw sequence files were deposited in the GSA database (https://bigd.big.ac.cn/gsa/), with the accession number of CRA002467. Other raw data supporting the conclusions of this article will be made available by the authors, without undue reservation, to any qualified researcher.

## Ethics Statement

The studies involving human participants were reviewed and approved by General Hospital of Guangzhou Military Command PLA, Sun Yat-sen Memorial Hospital, the First Affiliated Hospital of Sun Yat-sen University. The patients/participants provided their written informed consent to participate in this study. The animal study was reviewed and approved by the Ethics Committee of the School of Life Science, Sun Yat-sen University. Written informed consent was obtained from the individual(s) for the publication of any potentially identifiable images or data included in this article.

## Author Contributions

CD carried out majority of the experiments. YL and SG carried out sequencing experiments and bioinformatic analysis. CS assisted with sequencing. HL assisted with part of the experiments. LD and SW provided patient samples and clinical discussion. JS provided medical imaging and analysis. KL provided discussion and language polishing. YC supervised experiments and wrote the manuscript. LW supervised the sequencing experiments and bioinformatic analysis. LL conceived the study, supervised all experiments, and wrote the manuscript.

## Conflict of Interest

The authors declare that the research was conducted in the absence of any commercial or financial relationships that could be construed as a potential conflict of interest.
