# Supplementary material for: Identification of Specific Joint-Inflammatogenic Cell-Free DNA Molecules From Synovial Fluids of Patients With Rheumatoid Arthritis
Source: Front Immunol. 2020 Apr 28;11:662. doi: 10.3389/fimmu.2020.00662 (PMC7198838; doi:10.3389/fimmu.2020.00662)
Supplement: Supplementary file 1 [file Data_Sheet_1.docx]

**Appendix A. Supplementary data**

**
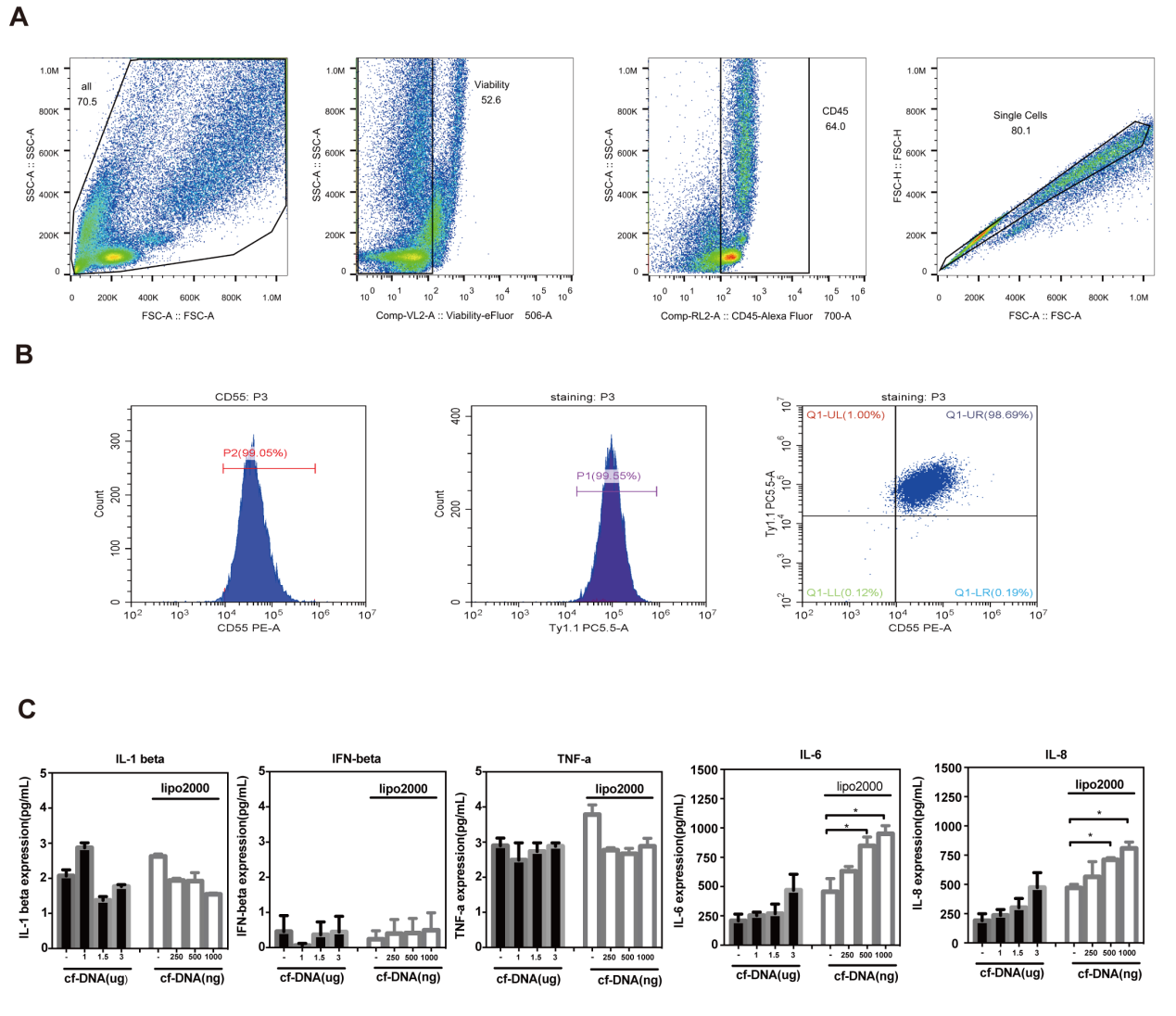
**

**Fig.** **S1. Analysis SFcfDNA stimulation with different cells. (A)** Gating strategy for the assay, briefly: after SFMICs were challenged with SFcfDNA and stained with Dead/live cell dye, CD45+ antibody, TNF-α antibody, the positive cells were gated. **(B)** Verifying primary FLS. Primary single cells purified from synovium tissues after passed 3 generations stained with CD55 and Thy1 antibody. From left to right: CD55 single positive percentage; Thy1 single positive percentage; double positive cells. Results obtained with CytExpert analysis software. **(C)** Cytokine expression of FLS after SFcfDNA stimulation. Cytokines beads assay exhibited that only IL-6 and IL-8 expression up-regulated. Statistical significance was calculated by unpaired T test, * *P*<0.05. Data are presented as the mean with S.D.

**
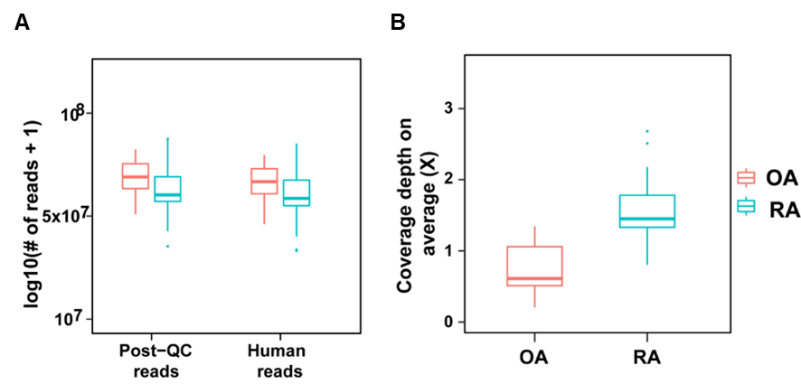
**

**Fig. S2. Sequencing summary.** (A) Distribution of reads after quality control (QC) and human reads used for analysis grouped by RA and OA. The y axis is shown with a logarithm scale. (B) Distribution of the average coverage depth on the whole human genome grouped by RA and OA.


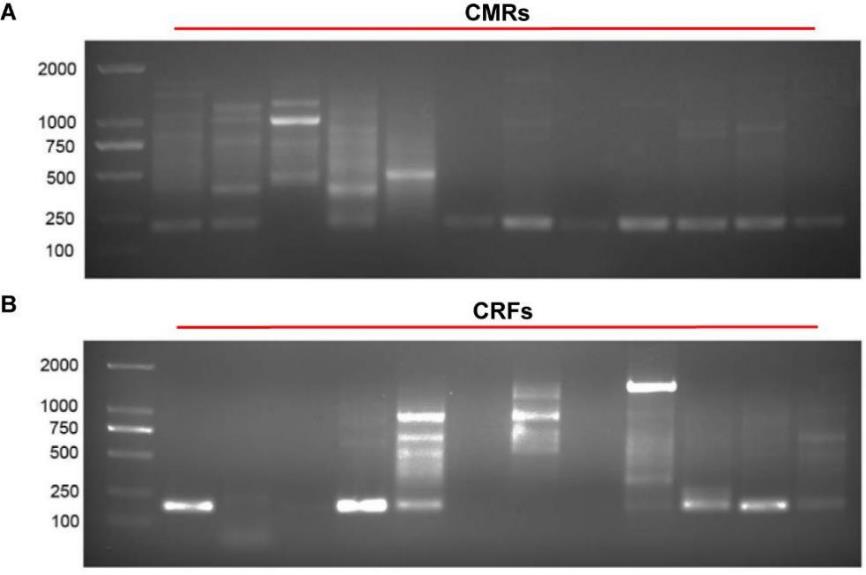


**Fig. S3. High frequency of CMRs and CMFs in SF of RA patients. (A)** 200 bp length of CMR and **(B)** CMF sequences obtained from sequencing.


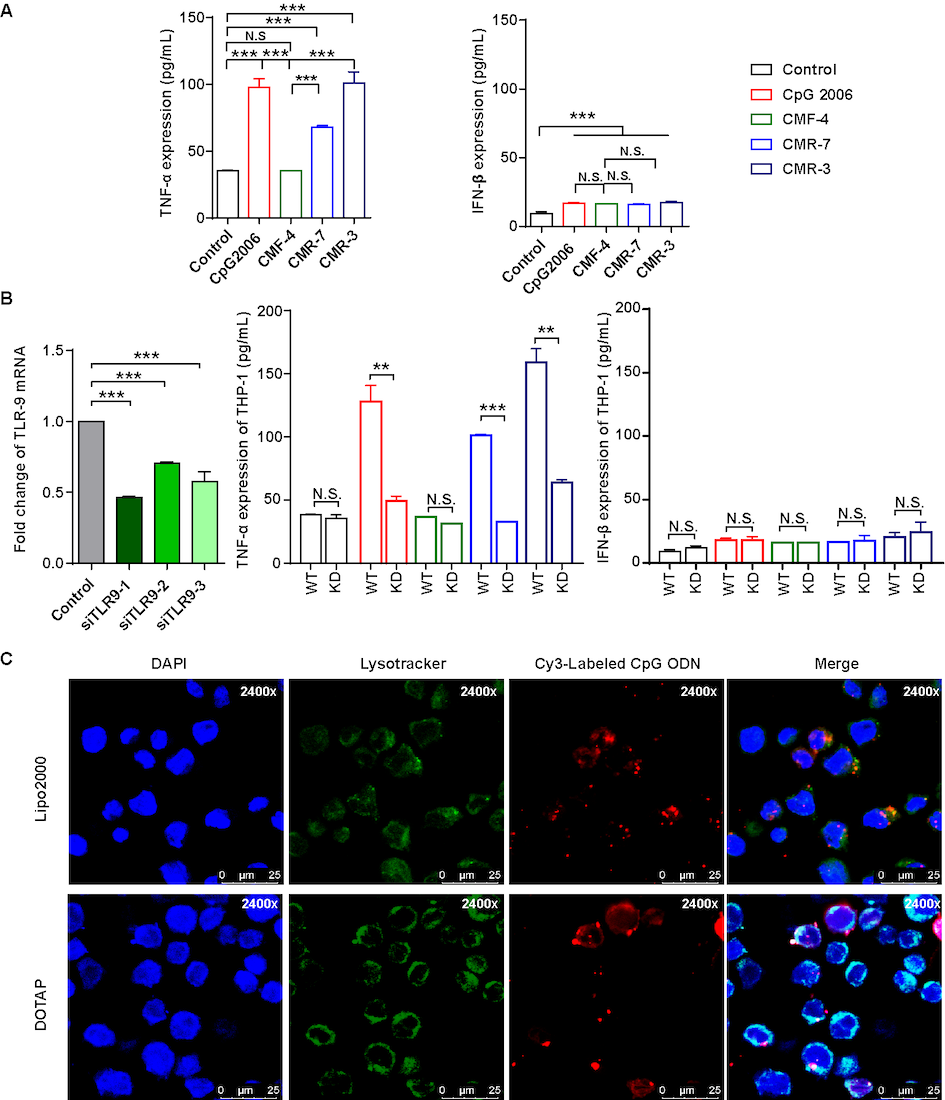


**Fig. S4. CpG-motif rich sequences activate cytokine expression via TLR-9 dependent pathway.** (**A**) TNF-α and IFN-β expression of THP-1 with CpG-motif rich sequences (CMR-3 and CMR-7), CpG 2006 and CpG-motif free sequence (CMF-4). (**B**) TNF-α induction obviously declined in THP-1 cells after siTLR9-1 introduction to the cell. There was not much alteration of IFN-β expression before or after TLR-9 knocking down. (**C**) Lipo2000 and DOTAP loaded DNA sequences were trapped into lysosome. Cy3-labelled CpG 2006 (red fluorescence) partially merged with lysosome stained with dye LysoTracker (green fluorescence) after 6 hours transfection. Statistical significance was calculated by Two-tailed paired T test, * 0.01<*P*<0.05, ** 0.001<*P*<0.01. Data are presented as the mean with S.D. The data shown are representative of two independent experiments with similar tendency.


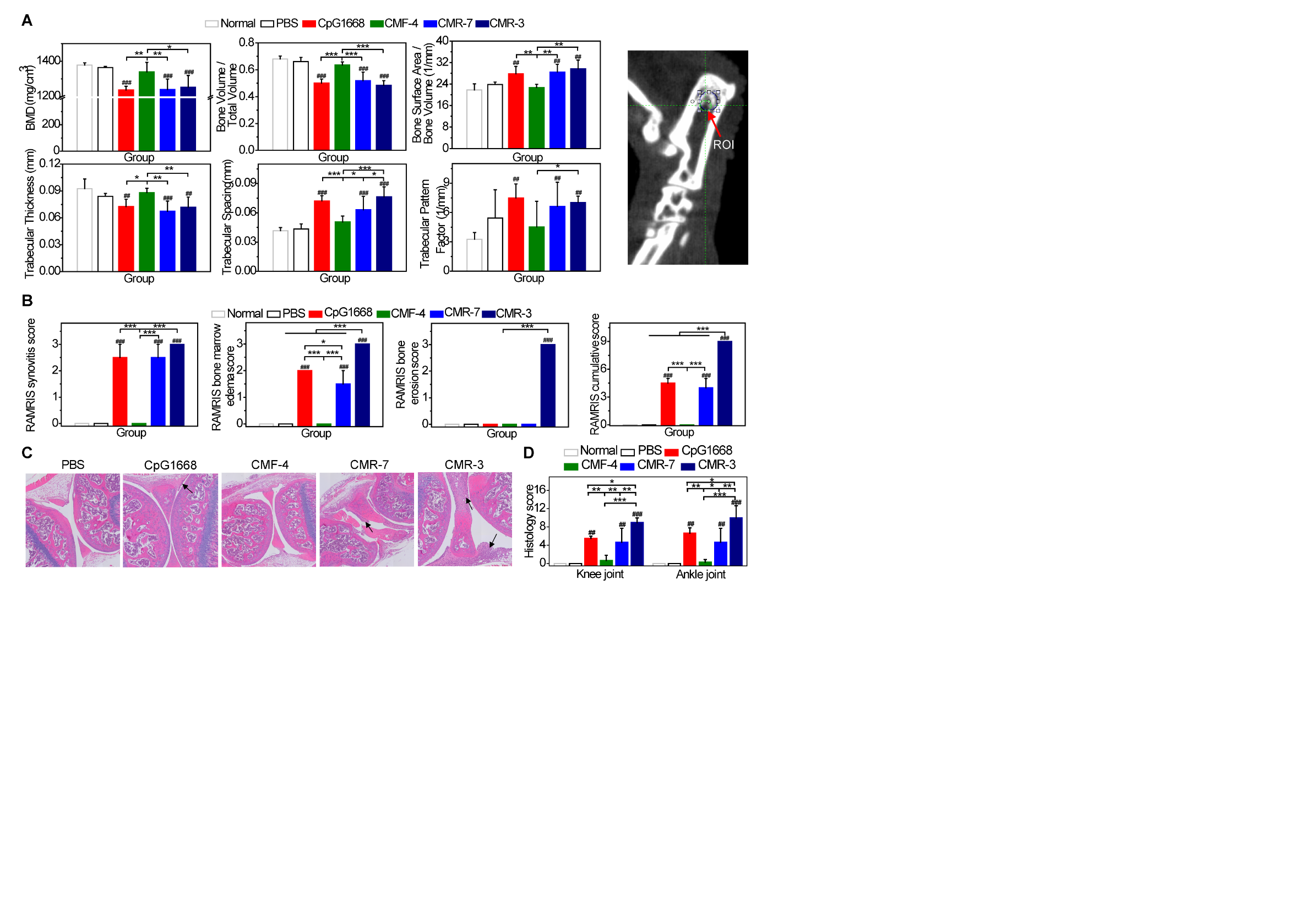


**Fig. S5. Iconography and histology study of mice with high frequency CMR sequences.** (**A**) Analysis of microCT data of ankle joint bone histomorphometric parameters. The bone trabecular of calcaneus was chosen as region of interest (ROI). BMD and the trabecular parameters, such as bone volume/total volume, bone surface area/bone volume, trabecular thickness, trabecular spacing and trabecular pattern factor were measured by Inveon Research Workplace. (**B**) Assessment by MRI of inflammation and damage of mouse knee joints using the Rheumatoid Arthritis MRI Scoring (RAMRIS) system. Additionally, the cumulative score as the sum of synovitis, bone marrow edema, and bone erosion scores is shown. (**C**) Representative H&E staining of the knee joints of mice 6 days after administration (×200). Inflammatory cell infiltration is indicated with black arrows. (**D**) Average histology scores of knee joints and ankle joints of mice 6 days after administration. In A, B and D, statistical significance was calculated by one-way ANOVA with the LSD post-test, ## 0.001<*P*<0.01, ### *P<*0.001 versus normal group. * 0.01<*P*<0.05, ** 0.001<*P*<0.01, *** *P*<0.001 between two groups. Data are presented as the mean with S.D.

**Table S1. Demographic characteristics of patients**

|  | HD  plasma | OA  SF | RA  plasma | RA  SF | RA  synovium tissue |
| --- | --- | --- | --- | --- | --- |
| Total subjects enrolled | 50 | 33 | 63 | 80 | 5 |
| **Methodology used** | | | | | |
| **Quantitation of cfDNA concentration** | | | | | |
| Subject No. | 50 | 33 | 63 | 80 | --- |
| Female sex | 68% | 65% | 69% | 74% | --- |
| Age (y) | 52 | 63 | 59 | 61 | --- |
| Virus infection | --- | --- | --- | --- | --- |
| **Intracellular TNF-α staining** | | | | | |
| Subject No. | --- | --- | --- | 10 | --- |
| Female sex | --- | --- | --- | 86% | --- |
| Age (y) | --- | --- | --- | 54 | --- |
| Virus infection | --- | --- | --- | --- | --- |
| **ELISA of cytokines** | | | | | |
| Subject No. | --- | --- | --- | 5 | 5 |
| Female sex | --- | --- | --- | 90% | 80% |
| Age (y) | --- | --- | --- | 52 | 54 |
| Virus infection | --- | --- | --- | --- | --- |
| **Sequencing and bioinformatics** | | | | | |
| Subject No. | --- | 23 | --- | 50 | --- |
| Female sex | --- | 100% | --- | 100% | --- |
| Age (y) | --- | 51 | --- | 48.5 | --- |
| Virus infection | --- | --- | --- | --- | --- |
| **Templates for specific sequences PCR (Mixed)** | | | | | |
| Subject No. | --- | --- | --- | 10 | --- |
| Female sex | --- | --- | --- | 100% | --- |
| Age (y) | --- | --- | --- | 55 | --- |
| Virus infection | --- | --- | --- | --- | --- |
| **Methylation assays** | | | | | |
| Subject No. | --- | 25 | --- | 50 | --- |
| Female sex | --- | 95% | --- | 80% | --- |
| Age (y) | --- | 59 | --- | 58 | --- |
| Virus infection | --- | --- | --- | --- | --- |

**Table S2. Antibodies used in flow cytometry**

| **Antigen fluorophore** | **Manufacturer** | **Clone ID** | **Source** | **Isotype** | **Dilution** |
| --- | --- | --- | --- | --- | --- |
| AF700 Human CD45 | BioLegend | 2D1 | mouse | IgG1, κ | 1:100 |
| BV421 Human TNF-α | BioLegend | MAb11 | mouse | IgG1, κ | 1:100 |
| PE Anti-Human CD55 (DAF) | BD PharMingen | IA10 | mouse | IgG2a, κ | 1:100 |
| PerCP-Cy5.5^TM^ Anti-Human CD90 (Thy1) | BD PharMingen | 5E10 | mouse | IgG1, κ | 1:100 |

**Table S3.** **qPCR Primers and siRNA duplex sequences used in the study**

| **Target** | **Primer sequences** |
| --- | --- |
| hTLR-9 | Forward: 5’- CTTCAATTACCAAAAGAGGGTGTC-3’ |
|  | Reverse: 5’-AAGTTCATCTGCAGACGCAGAG-3’ |
| siRNA | TLR9-1: 5’- GCAAGCTTAACCTGTCCTT-3’ |
|  | TLR9-2: 5’-CTTCGTGGTCTTCGACAAA-3’ |
|  | TLR9-3: 5’-TACCACGAGCACTCATTCA-3’ |
| mTNF-α | Forward: 5’-CCCTCACACTCACAAACCAC-3’ |
|  | Reverse: 5’-ACAAGGTACAACCCATCGGC-3’ |
| mIL1-β | Forward: 5’-AATTGGTCATAGCCCGCACT-3’ |
|  | Reverse: 5’-AGAAGTCAATAGCCAGGCAGA-3’ |
| mIL-6 | Forward: 5’-ACAACGATGATGCACTTGCAGA-3’ |
|  | Reverse: 5’-GATGAATTGGATGGTCTTGGTC-3’ |
| mMMP-3 | Forward: 5’-ACTCTACCACTCAGCCAAGG-3’ |
|  | Reverse: 5’-TCCAGAGAGTTAGACTTGGTGG-3’ |
| mCXCL-1 | Forward: 5’-TGAGCTGCGCTGTCAGTGCCT-3’ |
|  | Reverse: 5’-AGAAGCCAGCGTTCACCAGA-3’ |
| mCXCL-2 | Forward: 5’-ATGCCTGAAGACCCTGCCAAG-3’ |
|  | Reverse: 5’-GGTCAGTTAGCCTTGCCTTTG-3’ |
| mIFN-α | Forward: 5’-GCCTTGACACTCCTGGTACAAATGAG-3’ |
|  | Reverse: 5’-CAGCACATTGGCAGAGGAAGACAG-3’ |
| mIFN-β | Forward: 5’-CCACCACAGCCCTCTCCATCAACTAT-3’ |
|  | Reverse: 5’-CAAGTGGAGAGCAGTTGAGGACATC-3’ |
| mβ-Actin | Forward: 5’-TGTGATGGTGGGAATGGGTCAG-3’ |
|  | Reverse: 5’-TTTGATGTCACGCACGATTTCC-3’ |
| hGAPDH | Forward: 5’- GTCTCCTCTGACTTCAACAGCG-3 |
|  | Reverse: 5’-ACCACCCTGTTGCTGTAGCCAA-3’ |

**Table S4. Number of sequencing reads for each sample**

| **Sample ID** | **Group** | **Raw Data** | **Quality trimming** | | **hg19 alignment** | |
| --- | --- | --- | --- | --- | --- | --- |
|  |  | **#** | **#** | **%** | **#** | **%** |
| **RA2_03** | RA | 41,861,754 | 39,880,708 | 100.00 | 38,262,733 | 95.94% |
| **RA2_04** | RA | 39,320,490 | 37,745,246 | 100.00 | 36,071,668 | 95.57% |
| **RA2_06** | RA | 37,099,680 | 35,224,472 | 100.00 | 33,557,627 | 95.27% |
| **RA2_07** | RA | 41,628,048 | 39,713,424 | 100.00 | 37,718,681 | 94.98% |
| **RA2_08** | RA | 39,066,624 | 37,277,306 | 100.00 | 35,551,930 | 95.37% |
| **RA2_09** | RA | 59,986,518 | 57,713,578 | 100.00 | 52,379,221 | 90.76% |
| **RA2_11** | RA | 65,872,194 | 62,472,252 | 100.00 | 57,650,468 | 92.28% |
| **RA2_14** | RA | 35,783,760 | 34,058,832 | 100.00 | 32,831,733 | 96.40% |
| **RA2_15** | RA | 51,399,928 | 49,418,752 | 100.00 | 47,618,545 | 96.36% |
| **RA2_17** | RA | 52,469,052 | 50,400,990 | 100.00 | 48,186,861 | 95.61% |
| **RA2_18** | RA | 40,185,100 | 38,578,030 | 100.00 | 36,936,331 | 95.74% |
| **RA2_19** | RA | 47,899,248 | 45,936,556 | 100.00 | 43,983,236 | 95.75% |
| **RA2_20** | RA | 48,106,898 | 45,972,490 | 100.00 | 44,250,660 | 96.25% |
| **RA2_21** | RA | 23,666,530 | 22,579,142 | 100.00 | 21,450,229 | 95.00% |
| **RA2_22** | RA | 62,528,444 | 60,074,896 | 100.00 | 57,648,447 | 95.96% |
| **RA2_23** | RA | 52,231,346 | 50,180,196 | 100.00 | 48,253,840 | 96.16% |
| **RA2_24** | RA | 53,861,992 | 51,728,456 | 100.00 | 49,795,231 | 96.26% |
| **RA2_25** | RA | 41,473,994 | 39,867,370 | 100.00 | 37,887,113 | 95.03% |
| **RA2_26** | RA | 33,736,426 | 32,166,858 | 100.00 | 30,697,968 | 95.43% |
| **RA2_27** | RA | 40,697,142 | 39,215,840 | 100.00 | 37,756,483 | 96.28% |
| **RA2_28** | RA | 37,204,930 | 35,728,218 | 100.00 | 34,297,465 | 96.00% |
| **RA2_29** | RA | 38,992,286 | 37,292,306 | 100.00 | 35,573,825 | 95.39% |
| **RA2_30** | RA | 41,717,224 | 39,707,968 | 100.00 | 37,839,922 | 95.30% |
| **RA2_33** | RA | 43,008,406 | 41,178,780 | 100.00 | 39,498,976 | 95.92% |
| **RA2_34** | RA | 49,533,790 | 47,546,774 | 100.00 | 45,342,249 | 95.36% |
| **RA2_35** | RA | 47,935,972 | 45,398,442 | 100.00 | 43,861,964 | 96.62% |
| **RA2_36** | RA | 49,830,460 | 46,890,922 | 100.00 | 44,585,606 | 95.08% |
| **RA2_37** | RA | 43,376,918 | 41,700,134 | 100.00 | 39,718,857 | 95.25% |
| **RA2_38** | RA | 40,126,986 | 38,329,694 | 100.00 | 35,865,202 | 93.57% |
| **RA2_41** | RA | 39,543,760 | 37,390,724 | 100.00 | 35,287,496 | 94.38% |
| **RA2_44** | RA | 32,285,946 | 30,705,200 | 100.00 | 29,102,176 | 94.78% |
| **RA2_46** | RA | 23,747,950 | 22,609,976 | 100.00 | 21,624,570 | 95.64% |
| **RA2_49** | RA | 54,727,714 | 52,717,502 | 100.00 | 50,426,897 | 95.65% |
| **RA2_50** | RA | 48,691,668 | 46,137,696 | 100.00 | 44,250,128 | 95.91% |
| **RA2_52** | RA | 54,044,742 | 52,270,362 | 100.00 | 49,870,565 | 95.41% |
| **RA2_54** | RA | 42,259,832 | 40,342,842 | 100.00 | 38,864,130 | 96.33% |
| **RA2_55** | RA | 50,719,974 | 48,346,502 | 100.00 | 46,436,207 | 96.05% |
| **RA2_57** | RA | 53,701,302 | 51,509,572 | 100.00 | 49,183,424 | 95.48% |
| **RA2_61** | RA | 39,646,518 | 38,147,060 | 100.00 | 36,551,412 | 95.82% |
| **RA2_62** | RA | 58,788,230 | 54,569,930 | 100.00 | 49,707,726 | 91.09% |
| **RA2_64** | RA | 47,016,670 | 44,204,672 | 100.00 | 42,291,378 | 95.67% |
| **RA2_67** | RA | 44,648,806 | 42,147,128 | 100.00 | 40,403,791 | 95.86% |
| **RA2_71** | RA | 40,180,592 | 38,131,124 | 100.00 | 36,286,476 | 95.16% |
| **RA2_72** | RA | 28,267,638 | 26,742,330 | 100.00 | 25,129,969 | 93.97% |
| **RA2_75** | RA | 30,181,580 | 28,386,910 | 100.00 | 27,065,858 | 95.35% |
| **RA2_77** | RA | 78,691,548 | 74,227,050 | 100.00 | 71,199,702 | 95.92% |
| **RA2_79** | RA | 39,594,192 | 37,157,268 | 100.00 | 35,791,498 | 96.32% |
| **RA2_80** | RA | 79,905,874 | 74,751,618 | 100.00 | 71,022,568 | 95.01% |
| **RA2_81** | RA | 24,522,830 | 22,540,128 | 100.00 | 21,735,898 | 96.43% |
| **RA2_82** | RA | 33,496,738 | 31,221,262 | 100.00 | 29,682,370 | 95.07% |
| **OA2_43** | OA | 34,475,512 | 32,313,620 | 100.00 | 28,922,183 | 89.50% |
| **OA2_47** | OA | 42,294,816 | 39,792,594 | 100.00 | 35,945,130 | 90.33% |
| **OA2_53** | OA | 41,220,954 | 39,236,772 | 100.00 | 31,332,741 | 79.86% |
| **OA2_60** | OA | 38,007,738 | 36,387,556 | 100.00 | 32,367,809 | 88.95% |
| **OA3_03** | OA | 84,642,874 | 66,676,326 | 100.00 | 62,432,136 | 93.63% |
| **OA3_06** | OA | 56,767,722 | 44,128,650 | 100.00 | 41,271,901 | 93.53% |
| **OA3_07** | OA | 62,169,790 | 46,458,196 | 100.00 | 44,351,317 | 95.47% |
| **OA3_08** | OA | 62,271,804 | 50,596,774 | 100.00 | 47,814,413 | 94.50% |
| **OA3_09** | OA | 81,721,700 | 64,573,008 | 100.00 | 61,253,540 | 94.86% |
| **OA3_10** | OA | 71,129,040 | 55,882,526 | 100.00 | 53,174,336 | 95.15% |
| **OA3_11** | OA | 61,578,366 | 49,001,356 | 100.00 | 46,476,492 | 94.85% |
| **OA3_12** | OA | 62,748,622 | 48,164,430 | 100.00 | 43,905,980 | 91.16% |
| **OA3_13** | OA | 61,044,004 | 48,097,414 | 100.00 | 43,604,064 | 90.66% |
| **OA3_14** | OA | 49,610,626 | 39,145,408 | 100.00 | 37,668,551 | 96.23% |
| **OA3_15** | OA | 52,498,974 | 41,944,072 | 100.00 | 40,054,672 | 95.50% |
| **OA3_16** | OA | 68,288,960 | 54,353,038 | 100.00 | 50,966,432 | 93.77% |
| **OA3_17** | OA | 73,672,186 | 59,012,862 | 100.00 | 55,496,599 | 94.04% |
| **OA3_18** | OA | 73,644,842 | 60,637,598 | 100.00 | 57,192,354 | 94.32% |
| **OA3_19** | OA | 69,690,530 | 57,193,874 | 100.00 | 54,141,465 | 94.66% |
| **OA3_20** | OA | 71,088,258 | 56,306,870 | 100.00 | 53,392,188 | 94.82% |
| **OA3_22** | OA | 65,354,552 | 53,793,318 | 100.00 | 51,292,715 | 95.35% |
| **OA3_23** | OA | 59,265,544 | 48,214,068 | 100.00 | 45,535,173 | 94.44% |
| **OA3_24** | OA | 71,686,702 | 58,146,680 | 100.00 | 55,584,133 | 95.59% |

**Table S5. Primers of high frequency CMRs and control CMFs**

|  | **PCR Length** | **Primers** |
| --- | --- | --- |
| **CpG-motif rich sequences (numbers of CpG≥20)** | | |
| CMR-1 | 200 bp | F:5’CCTTGGGCGCCCCCAGCCGGGA3’  R:5’CGGGGCCGAGGTCAACTGGGCG3’ |
| CMR-2 | 200 bp | F:5’CGCCTCTGTGGCGGCTGGGCCC3’  R:5’CGGGGGCACAAACCCGGGACC3’ |
| CMR-3 | 200 bp | F:5’GAGACCGATCCCTCAGGAGCCG3’  R:5’AGAACGCGTCTGGGCCGACGAGC3’ |
| CMR-4 | 200 bp | F:5’GGCCCAGCCTAGCCCAGCGGG3’  R:5’CCGAGGCCGAGGTGCTGGAGCA3’ |
| CMR-5 | 200 bp | F:5’CAGTGAGGAGAGAGGGCGGGGC3’  R:5’CCCCGCGCCCTCTCTGCCTCTCCTC3’ |
| CMR-6 | 200 bp | F:5’ACTCTCGCTGCTACTAGGGGAG3’  R:5’AAAGCAAGTTCGAGCGTAAAAG3’ |
| CMR-7 | 200 bp | F:5’GCCCACGGGCGGCCAATTGCCGG3’  R:5’CTGCGCTCTCCTGGCTGCTGGACC3’ |
| CMR-8 | 200 bp | F:5’CTTCGCGGTACCCGGACAGAT3’  R:5’TGGTTCACCTCAAGGAACTCG3’ |
| CMR-9 | 200 bp | F:5’GTCCCCCAAACCTCGTCCTCCGG3’  R:5’ACACCGGCTGTGGCGCTGCTTTAC3’ |
| CMR-10 | 200 bp | F:5’GAGACCGATCCCTCAGGAGCCG3’  R:5’AGAACGCGTCTGGGCCGACGAGC3’ |
| **CpG-motif rich sequences (numbers of CpG<20)** | | |
| CMR-11 | 200 bp | F:5’ATCCGTGGCAAACAACATATG3’  R:5’ATT GTT TTC AGC GGT GCA AT3’ |
| CMR-12 | 200 bp | F:5’ACTCTCGCTGCTACTAGGGGAG3’  R:5’AAAGCAAGTTCGAGCGTAAAAG3’ |
| CMR-13 | 200 bp | F:5’CTTCGCGGTACCCGGACAGAT3’  R:5’TGGTTCACCTCAAGGAACTCG3’ |
| CMR-14 | 200 bp | F: 5’GTCCCCCAAACCTCGTCCTCCGG3’  R: 5’ACACCGGCTGTGGCGCTGCTTTAC3’ |
| CMR-15 | 200 bp | F:5’TGCCAGCTCTGCCCACTCTAAGG3’  R:5’ATCCCTGGCCAGGGGTGAGGGG3’ |
| CMR-16 | 200 bp | F:5’CGGATTGGACATTCGGAAGAGGG3’  R:5’GCACCTCCGCCATCTTCGGCAGC3’ |
| CMR-17 | 200 bp | F:5’CCAGGGGGCATTTGAAGGGC3’  R:5’TCCGGCGCCCCAGGAGGAGG3’ |
| CMR-18 | 200 bp | F:5’AATCCCGGCCTGCTCCGCCCGG3’  R:5’GGCAGACGACCCCTGCCCGT3’ |
| CMR-19 | 200 bp | F:5’CGCGCTTTCCTCAGAGGCTCGG3’  R:5’GCTTCCGCTCTCTCCGGGCAACC3’ |
| **CpG-motif free sequences** | | |
| CMF-1 | 200 bp | F:5’GACAACCAATACATAAATGAATG3’  R:5’CAGTACCAGTGAATATGGTAAGT3’ |
| CMF-2 | 200 bp | F:5’GTAGTGATACAGATGTGGGATG3’  R:5’GTATCACTACATATCTGTATCA3’ |
| CMF-3 | 200 bp | F:5’CAGTTTTCTCATCCAACTAAGG3’  R:5’ATAAACCATAAGTCAGTTACACA3’ |
| CMF-4 | 200 bp | F:5’CAGGGTGATAGTTACATGGATGG3’  R:5’CTACCATCTACATATCTACTA3’ |
| CMF-5 | 200 bp | F:5’CGGATTGGACATTCGGAAGAGGG3’  R:5’TGCACCTCCGCCATCTTCGGCAGC3’ |
| CMF-6 | 200 bp | F:5’CATTTAGTGTGTCAAAGGAAC3’  R:5’TGGGGTGAGAATAATGGGA3’ |
| CMF-7 | 200 bp | F:5’CATGTTTGTGAAGATCTTAAAAG3’  R:5’CTTTACTTTTAATACATATCAAGT3’ |
| CMF-8 | 200 bp | F:5’CTCTATCCACCCTGTTATTC3’  R:5’ATAGTATGAGATAGTTTTGGA3’ |
| CMF-9 | 200 bp | F:5’CCCACCTACCCCACACATGAAA3’  R:5’TAGGTGGGGATCTGTGGGATTGG3’ |
| CMF-10 | 200 bp | F:5’CAGTGAGGAGAGAGGGCGGGG3’  R:5’TCCCCGCGCCCTCTCTGCCTCTCC3’ |

**Table S6.** **PCR product information of high frequency CMRs and control CMFs**

|  | **Chromosome location** | **Original length** | **Sequences information** |
| --- | --- | --- | --- |
| **CpG-Motif rich sequences (numbers of CpG≥20)** | | | |
| **CMR-1** | Chr1 | 1383 bp | CCTTGGGCGCCCCCAGCCGGGACCGAACGTGGTCCCCACCGCGGACAGGCGACCCTTGTGTGGGCGCCACAGAAGATGCAAACGCGACTGTGCACCTGTGTCCTGCGCGCAGCCCCCGTGGCTCGCTCACCCCTAGCACGGCCTGCACCAGCGGCGTCTTGCCCTCATCCTCCGCGTCCGCCCAGTTGACCTCGGCCCCG |
| **CMR-2** | Chr8 | 1449 bp | CAGGGCAGCACCGCAGCGGTCCGCAGCGGACAAGGTCTCCTGCAGCCGCAGCAGGGAGCAACGCACACCGGCTCCGGAGCCCGCCATGCCGCCGAGTCCCGCTCCCGCGCGTGCGCCCGCTCGGCCCGGACCCGGAAATGCCCCTACGCGCGGAGGCGGGGCTGCGGGGCGCGGCGGCAGGAACTTTCCCGGGGACCCCT |
| **CMR-3** | Chr1 | 237 bp | CGCCTCTGTGGCGGCTGGGCCCCGCGGGGTCGGGGGGTGGGGGTGCCCAACGTGGCAGGGAGGCCTGCAAGGGGAGGCCGAGGCGCCGGTGGAAGCACAAACCACCCGGCGAGTCAGAGCAGGGGTCGGGGCCCCTGGCCGCACCGTGCCGCGCATGCGCAGGGTCCCGGGGTCCCTGGGGTCCCGGGTTTGTGCCCCCG |
| **CMR-4** | Chr7 | 851 bp | GGCCCAGCCTAGCCCAGCGGGTGGAGGGCGCACCTCGTGCTGGGCGCCGGGGAAGCGCTCGCGCACGCGCCGGAAGAGCTGGCGGAAGGTGGCGCGCACCACGGCGGGGCACGCGGGAACCGAGCGGCTGAGCGCGCTCAGCAGGGCCCCCAGGTGGGCGCGCAGCGTCTGCGCGCTCTGCTCCAGCACCTCGGCCTCGG |
| **CMR-5** | Chr19 | 530 bp | GAGACCGATCCCTCAGGAGCCGCGGGCGAAAGCCCACCCGACGCTGGCGACGAGCACAGACACCTCAGCCGCCGCAGCCATCTTGGCACATCCGGCTCGGGTCTCCGCGGCCGCCGCGCCGCCGACGTGTCCGGCTTACGACATCAGCGCGCGCCCCGCCCCTGGCCCCGCCCCTCGGCTCGTCGGCCCAGACGCGTTCT |
| **CMR-6** | Chr11 | 1033 bp | ACATGGTGAGTCTTACTGTTGCGGGCTCCGGGGCCGTCGACCATGCCGCTCGACCTCCACCTCCGCTGGGAAGCTGAGGCGCCAAACGGCTCCCAGAGGGTCCCGGGAAGCGCATGGTGAGGGTCCCCGGGCCGGCTGTGCAGCGGAATCGGGCCCTTCCCGGACTCGCGCCCTTCCCGGACTCGCCGCTGGCGAGCGCG |
| **CMR-7** | Chr3 | 1159 bp | GCCCACGGGCGGCCAATTGCCGGCGCTCCCCGCGCGGCTCTGAGCGCCCCGTCCCGCCGGCGGCCGCGAGACCAGAGCGAGCGAACGAACCGCGGCGGTCCGGAGAGCCCCGAGCGCAGCGCAGGACCTGGGTACGCCGCGAGGAACCGTGCAGCCCAGCGCGGCCGCCCGGCCCGGGTCCAGCAGCCAGGAGAGCGCAG |
| **CMR-8** | Chr2 | 1205 bp | GGCGAGTTGGCCGCGTGTGTGCCTCGCTGTTTGACGCGAAGACGAGCCAATCAGGGCGGGCGGCCCGAGCTGCCATGTGACGGGCAAGGCGGCCCCTTTCCCCAGCGCGGCCAGAGGGAGGAGAGAACCGGGGCTCGCCGCGAGCCTTCGAGAGCAGCGGCCGCGGAGGAGGCGGCGGCGGCGGGCGGGAGCAGCGGCGG |
| **CMR-9** | Chr8 | 1457 bp | CGGTGGCCCCGCGGAGTGTGGGGACTCAGGGTGGGGGTGCGAGGAGAGGCGCGCGCCCCAGCGGCCGTCCGGGGGCGGAGGGGGAAGGGAGAGAGCCGGCCAAGGCGGGGCCCTCCCGCGCGCGCGTCCTCCGCGCGCCCCCACCCCGTGCGGCGCCGAGCTCTCCACGCCCAGACCCTGCTACTCCACTGGGGTCCGAC |
| **CMR-10** | Chr10 | 1580 bp | CGATGCCGGGCGGCCGGAGCCATTGACCCGGGACGCCGCCGTCCGCTGAGCAGCCGACCACCCCGCCGCCTCCGGTGCATGGGGACTGGCTGAGGAGCCAGCATGGGCAACTGCGTGGGGAGACAGCGCCGGGAGAGGCCGGCAGCCCCGGGACACCCCCGCAAGCGAGCAGGTAACGATGGGGAAGGGAGCAGGGCCTC |
| **CpG-Motif rich sequences (numbers of CpG<20)** | | | |
| **CMR-11** | Ch12 | 1012 bp | ATCCGTGGCAAACAACATATGCAAACGACATATGCAAACAACATATGCAAACGATTATCTATGTGCAGATAATTCCATGCCCACGAGCCGACAGATTTGCAATTCTCAAGTACTTCTATGTGCATGTAATTCTACGGGCAAATTGTTCCGAGTGCAAGGGATTTGGTGTGTGTGTCAGTGATTGCACCGCTGAAAACAAT |
| **CMR-12** | Ch11 | 1033 bp | ACTCTCGCTGCTACTAGGGGAGTCCACGCCTCCACCTTTTCCCCGCCTCCTGTAACTCATTGGAAGAGCTGTGCGCCAATTACTTTGCCGTTACCCAATGGCTACTCAACTGTCTCTTTGGTTCCGCCCAGTCGTCTACCCTGGGTTGGCAACGCCTGGGTCTACGCATGCGCCCAAACGCTTTTACGCTCGAACTTGCTTT |
| **CMR-13** | Ch19 | 304 bp | CTTCGCGGTACCCGGACAGATGTCTCCACACTCCTCGTTGTCATCTTTGTTCAACACGATGTAATTATCCTCCACGGAATCCAGGATACGGGACCAGTCGATAGTGGCCAAGTAACAGAGCTCATTGTTCTTCTCGATGCGGACAGAACCCCGGGTGATGTTCATCAGGTTGTAGAGGCCGAGTTCCTTGAGGTGAACCA |
| **CMR-14** | Ch2 | 763 bp | GTCCCCCAAACCTCGTCCTCCGGGACTATGTCTTCTCGCGGTCTCTACCCACCCGGGCCTGTGAAGGTCTTGCCGGATCCCGCCTAACAAAAGTTACCTCCTCTCCTGAACTCCCCTAAGGCACACCTGGGCAGAGGTGTTAGGAAGCGGAGGGTCCGGCCTGGGGTCTTCCGCACGTAAAGCAGCGCCACAGCCGGTGT |
| **CMR-15** | Ch19 | 2081 bp | TGCCAGCTCTGCCCACTCTAAGGTCCCGCCCACTTCCACTCCTTGGGGGCGGCACCCTCCCCTTGGTCCTGTGGGCCCGTTCTCCAGCAGAAAACCACGCCCACCAAGCAGAGGCCACGCCCACAACCGAAGTCAACGCCAACCCTGTACTCAAACCTCGGCCCATAGTTCCTCAGATCCCCTCACCCCTGGCCAGGGAT |
| **CMR-16** | Ch19 | 530 bp | CGGATTGGACATTCGGAAGAGGGCCCGCCTTCCCTGGGGAATCTCTGCGCACGCGCAGAACGCTTCGACCAATGAAAACACAGGAAGCCGTCCGCGCAACCGCGTTGCGTCACTTCTGCCGCCCCTGTTTCAAGGGATAAGAAACCCTGCGACAAAACCTCCTCCTTTTCCAAGCGGCTGCCGAAGATGGCGGAGGTGCA |
| **CMR-17** | ChX | 1243 bp | CCAGGGGGCATTTGAAGGGCCTCTGGCCCTGGCTGCTGCTGTGCGTCAGCAGGTGCATCTTCAGCTGGTGCTTCTTGGCGAAGGTTTGCCCGCACAGCGCCTCGGGGCACAGGTACAGCACCACGCCTGGACCAGAGCCCAGCAGTCCGCGGGGGCCCAGGGCGGCGGCCAGGCCCTCCGCCTCCTCCTGGGGCGCCGGA |
| **CMR-18** | Ch2 | 881 bp | AATCCCGGCCTGCTCCGCCCGGCGCCCGCGGCCCCTCACCTGTAGAAGGCCGCGTTGACCCTCTTTTCGCTGGGGAAGCCCAGCATCCCGCAGACCACGTGGCTGTTGTGGGCGCTCCAGCCTTTGTCGCACACTTGCGACCAGCCGTCAGGAAGCCTGACTTCCACCAGCCCCTCCGTCACGGGCAGGGGTCGTCTGCC |
| **CMR-19** | Ch15 | 474 bp | CGCGCTTTCCTCAGAGGCTCGGCCGGGCGCCCTGCTTCGAAGTGGGCCTGAGGAAGCCTCCGCCTCCGCCGCTGCTTTCTCCCCCTTCCTTCTCCAGCGGCTCCTCCAGGCCGCTCCAGAGACCCCGAGGACCCAAGGACGGTGCCGGGCGGAAAGTCTGTGCGAAGCTGGTGAAAAGGTTGCCCGGAGAGAGCGGAAGC |
| **CpG-Motif free sequences** | | | |
| CMF-1 | Chr10 | 200 bp | GACAACCAATACATAAATGAATGAACTATTTCATATTCTGATATAAAACTCCTCTCTCTCTTTTTTACATCCACAAATAAATTTTAAGTTTCCCCACTCATATTCACTCTGTACCATGTAGTTATTTCTCTCTCTATTTTTTCCTTACTCCCATGTAAATCATCAAATTCTTTATGTACTTACCATATTCACTGGTACTG |
| CMF-2 | Chr6 | 200 bp | GTAGTGATACAGATGTGGGATGATACAGATGTGTAGTAATACAGGTGTGGGATGATACAGATGTGTAGTAATACAGGTGTGGAATGTACAGATGTGTAGTGATACAGGTGTGGGATGATGTAGATGTGTAGTGATACAGATGTGGGATGTACAGTTGTATAGTAATACAGGTGTGGAATGATACAGATATGTAGTGATAC |
| CMF-3 | Chr6 | 200 bp | CAGTTTTTAAGAGTACTTTGTATGATAGGGATATTATATTAGTCCTTGGTGATCCTTTGTATGGTAGGGGTATTATATTAGTCCTTGGTGATATTTTGTATAGTAGGGGTATTATATTAGTCCTTGGTGATCCTTTGTATGGTAGGGGTATTATATTAGTCCTTGGTGATATTTTGTATGGTAGGGGTATTATATTAGTC |
| CMF-4 | Chr20 | 200 bp | CAGGGTGATAGTTACATGGATGGTAGTTGTTTGAGGTGTAGTTACATGGGGTAGTAGATATTTGGGGTGGTAGTTACAGAGGTTGGTAAACACACAGGGTGGTAGTTACATGTGGTGGTAGTTACATGGGATAGTAGTTACACAGAGTGATAGTTACATGAGGTGTTAGTTATTTGGGGTAGTAGATATGTAGATGGTAG |
| CMF-5 | Chr6 | 200 bp | TGATGGTTGGTTCCTGTTCTGATGGTTGGTTCCTGTTCTGATGGTTGGTTCCTGTTCTGATGATTGGTGTCCTGTTCTGATGGTTGGTTCCTGTTCTGATGGTTGGTGTCCTGTTCTGATGGTTGATTCCTATTCTGATGGTTGGTTTCTGTTCTGATGGTTGGTTCCTGTTCTGATGGTTGGTACCCCCTGTTCTGATG |
| CMF-6 | Chr4 | 200 bp | CATTTAGTGTGTCAAAGGAACAAGATTATCAATCATTGAACACATATAACAATTTCTACTTGTCCACTCATCACTATAATTTTCAAGTGTTCATTTCATCTCATATACTATTTCCAGACTTCAGAGTTATTCCTTTAGATTCCCCTTTCAACTGACTCATATCCATCCTTTATTCCAGTTTTCCCATTATTCTCACCCCA |
| CMF-7 | Chr8 | 200 bp | CATGTTTGTGAAGATCTTAAAAGTTTGGGTACCATCTTCTTATGATTAATGAAATCAAGAAAGGTGGTAACACAAAAACATTTCAGGTGAATATGTTAGTAGGATCAGAGGTTGAAATGGTGATGTAGAGAAGGAATTCAAATTATTCTGAATTTCTAAATCTAGGAAAATAAATAACTTGATATGTATTAAAAGTAAAG |
| CMF-8 | Chr14 | 200 bp | CTCTATCCACCCTGTTATTCACAAGAAATATTCTCAAGTACAATAGTAAGAAGAAGTTCCAAACCAGATTTAACATTTCACAGAGTTAAACACACTGTGTTGAGGACCTATTACAGTTTACTCTAATACATGTTTGGGTTGTCTGTCAAACATTTCCTGACACTGTATTAGTGAGAAAGTCCAAAACTATCTCATACTAT |
| CMF-9 | Chr5 | 200 bp | CCCACCTACCCCACACATGAAAACCAATCCCACAGATCTCCACCTACCCTACACATGAAAACCAACTCCACAGATCCCCACCTACCCCCACACATAAAAACCAACTCCACAGATCCCCACCTACCCCACACATGAAAACCAATCCCACAGATCCCCACCTACCCCACACATGAAAACCAATCCCACAGATCCCCACCTAC |
| CMF-10 | Chrx | 200 bp | CATAGGGAAAATAGTAAGAGACAAGTTTCAAACTGTTTCTGGTAGGTCAAACACAAAATTAGTCCATGATACTTCCAACACCTCAAGAGGTAGGGAAGAACTCCATTTTCCTTTTTTGTTCCCTCCTCTCTAGAGTACTTTGGGGGATCTTAAAACTCAAAACATAAGACTTAATCATTCCCCAATGTTTTATCAAATAG |
